# Quacks and the Bounds of Human Credulity

**Published:** 1914-08-15

**Authors:** 


### THE PROFESSION OF MEDICINE.

### Quacks and the Bounds of Human Credulity.

In a recent issue (The Hospital, May 30, p. 252) attention was drawn to the efforts of an individual doctor in the United States to stem the tide of sexual quackery, which seems to have risen in that republic to a higher level than in this country. It deserves, however, to be recognised that departmental action to mitigate the evils of advertising quacks and humbugs is much more energetic over there than with us. Two interesting examples of the difficulties which beset the eradication of this sort of fraud have just been furnished by Dr. W. J. Robinson in his usual trenchant, fearless style.* Inasmuch as they throw light on two of the most important factors in the vital processes of quackery, they are full of warning and instruction to the medical profession, and especially the public health services of this country.

A certain firm in America advertised a radium remedy for the cure of cancer. The attention of the authorities was attracted to the advertisements through the fact that the amount of radium claimed to be contained in it exceeded the entire quantity of that precious element believed to exist in the whole United States. An analysis was thereupon undertaken, and it turned out that the remedy was simply quinine and water, without the slightest trace of radio-activity at all. Accordingly, steps were taken to prohibit the sale of this fraudulent concoction, under powers which the Government departments have over swindles of this type. So far there is nothing of any exceptional note about the fraud, which seems to have been detected and stopped much more quickly and effectively than it would have been in this country. (Parenthetically we may add that it is quite probable that similar swindles are being perpetrated amongst us now with absolute impunity.) The sequel is what distinguishes the story. For a year and a half after the suppression of this radium imposition, the department was bombarded with requests from the dupes, begging that the quack vendors might be allowed to resume business, because they (the dupes) were convinced of the great benefits they had derived from the remedy. They considered it very cruel of the Government to deprive them of a medicine which was sure to make them well and save their lives!

Could anything illustrate better the bounds, or rather the boundlessness, of human credulity and folly? Can anyone wonder that quackery flourishes and is a power in the land? Is it astonishing that laws and Governments fail to quell it? But over and beyond this extraordinary trait of human nature, the cunning of the fraternity which exploits it is most ingeniously developed and employed. Let us quote an instance in point. The Department of Agriculture under the (U.S.) Food and Drugs Act has recently been investigating a new dodge of the secret remedy and proprietary medicine mongers. Advertisements are inserted broadcast through the press purporting to be written by a member of the lay public who has been saved from death in one of a number of specified serious illnesses by a wonderful prescription ordered for him or her by a great specialist. The latter, it is affirmed, will not allow his name to be made use of, because that course is contrary to medical ethics. Feeling it a duty to mankind to communicate this valuable remedy to other sufferers, the advertiser offers to supply it free to anyone who sends a postcard with his name and address. Those who fall into the trap receive in return a prescription drawn up in the usual physician's form, containing a number of ordinary ingredients, and also a large proportion of some patent or proprietary drug. In order to make up this prescription, the chemist has to use the branded product as ordered, and in so doing purchases, of course, a supply of it from the manufacturers. The chemist, naturally, recoups himself for this outlay by charging a higher price for the bottle of medicine; so it is the consumer who pays in the long run, as, of course, the advertisers intend that he should.

The peculiar cleverness of this method is that the Government cannot reach those who resort to it, either under the Food and Drugs Act or the Postal Laws. The deception and misrepresentation appear in advertisements, circulars, or letters, all separate from any package of medicine; indeed, the latter never goes through the post at all. The best the Department can do is to warn people to be suspicious of those who spend money on advertising, postage, and letter-writing out of self-styled philanthropy and love of their fellowmen. So far, to the best of our knowledge, this ingenious aid to fraud has not been introduced into Britain.

* *Critic and Guide*, May, 1914.

